# When do democratic transitions reduce or increase child mortality? Exploring the role of non-violent resistance

**DOI:** 10.1016/j.socscimed.2022.115459

**Published:** 2022-12

**Authors:** Aaron Reeves, Laura Sochas

**Affiliations:** Department of Social Policy and Intervention, University of Oxford, United Kingdom

**Keywords:** Democracy, Child mortality, Protest, Civil war, Political economy

## Abstract

What explains variation across countries in the effect of democratization on child mortality rates? Democratic transitions, on average, improve health outcomes but there is substantial variation across countries in whether democratization leads to lower-than-expected child mortality post-transition. As yet, there is no convincing quantitative explanation for this variation. In this paper, we argue that whether you have a protest-led or violence-led democratic transition alters the trajectory of child mortality post-transition. Our paper makes two contributions. First, we offer a more detailed account of how the type of resistance movement promoting regime change affects health post-transition. We also draw on novel data to categorise the movements producing democratic transitions as violent or peaceful, moving beyond earlier work which operationalised peaceful democratizations in terms of battle-related deaths. Second, we extend earlier research by examining whether the nature of the democratization movement constitutes a *necessary cause* of higher or lower-than-expected child mortality following democratization. Across 51 transitions, countries that have a protest-led transition have lower-than-expected child mortality rates after the transition to democracy than countries with other kinds of movements (*β* = −0.17, *p* = 0.003). Countries with violence-led transitions, meanwhile, have, on average, higher-than-expected child mortality rates after their transition (*β* = 0.20, *p* = 0.001). These associations hold when we adjust for covariates (including all possible combinations of various confounding variables). We also find evidence that protest-led transitions may be a necessary condition for avoiding increased child mortality post-transition. Finally, we conduct a deviant case analysis of transitions that appear to be contrary to our theory, finding that these cases are likely instances of measurement error. Democratization may not always improve health, but such health improvements are more likely when regime change is protest-led. This is because such movements are more likely to build broad coalitions committed to consensual politics post-transition, a critical feature of successful democracies.

## Highlights

• The effect of democratization on child mortality varies across contexts.
• We argue that protest-led democratization lowers child mortality post-transition.
• Countries with protest-led transitions do, in fact, have lower mortality rates.
• Indeed, protest-led transitions may be a necessary cause of mortality reductions.

## 1 Introduction

Democracies tend to have higher life expectancy, lower child mortality and even higher well-being than non-democracies (Baker et al., 2018; Gerring et al., 2012; Gugushvili and Reeves, 2021), and many countries that have transitioned to democracies have seen mortality rates decline faster than might have been expected based on previous trends (Bollyky et al., 2019). However, this is not always true (Ramos et al., 2020). Indeed, in some contexts, such as Grenada or Suriname, democratization is actually followed by a period of higher-than-expected child mortality. Even more puzzling, many of the most obvious potential explanations for this variation, such as the level of economic development, the amount of corruption, or the type of political system which follows democratization, are largely uncorrelated with estimates of child mortality post-transition (Ramos et al., 2020). This paper takes up the unresolved question of the heterogeneous effects of democratization on child mortality and offers a potential explanation for this variation.

What, then, could explain this variation across countries in the effect of democratization on child mortality rates? Our argument is that the type of transition to democracy alters the trajectory of child mortality post-transition. All democratic transitions involve conflict, usually over who has the right to govern, and sometimes these conflicts can be violent. But conflict is not necessarily violent. There are a range of nonviolent forms of conflict, such as ‘boycotts, strikes, protests, and organised non-cooperation’ all of which can be used ‘to extract political concessions and challenge entrenched power’ (Chenoweth and Stephan, 2011). Our main hypothesis is that nonviolent forms of democratization generate lower-than-expected child mortality post-transition while violent forms of democratization create higher-than-expected child mortality. To understand why, we review the most common theoretical arguments for why democracy is expected to improve health and lower child mortality (representation; accountability; competence). We demonstrate that there is substantial variation in the extent to which actual democracies display these ideal “health-promoting” characteristics, and hypothesise that democracies with nonviolent democratic transitions are more likely to operate according to these ideal principles.

There are three broad explanatory mechanisms that link democracy and health in theory. The first concerns “*representation*”, or what might be called a dictatorship of the poor. Democracies are more likely to enfranchise low-income citizens (Ansell and Samuels, 2015). ‘Since widespread human misery is unpopular’ (Gerring et al., 2012: 2), voters' preferences incentivise democratic leaders to implement policies which will improve health (Kudamatsu, 2012), such as increased access to healthcare, including immunizations (Lake and Baum, 2001), clean water (Biser and Edwards, 2012), and primary education, especially for people in marginalised areas (Harding, 2020). In addition, a distaste for inequality is inherent in the ideology of democracies (Gugushvili and Reeves, 2021) and this ideology may empower oppressed groups to make claims on politicians to implement policies that will mitigate their oppression. The second mechanism is “*accountability”*. Democracies are assumed to have more mechanisms for citizens and civil society organizations to hold leaders to account compared to autocracies. This could be through elections, e.g., the electorate can ‘fire’ politicians (Kudamatsu, 2012), but it might also be through a free press (Wigley and Akkoyunlu-Wigley, 2017), which can hold politicians to account for failures to ensure human welfare. Crucially, democracies foster vibrant civil society organizations that ‘encourage better governance and greater attention to the needs of the less advantaged citizens in a society’ (Gerring et al., 2012: 3) by putting pressure on political leaders to deliver on their promises. These organizations have been crucial in ensuring that vaccination programmes reach deprived groups (Gauri and Khaleghian, 2002). The third explanation is *“competence”* (Besley, 2005). Although prominent counterexamples exist, there is evidence that democratic competition among candidates produces leaders who possess particular skills, such as coalition-building and administration (Acemoglu et al., 2010; Besley and Reynal-Querol, 2011; Galasso and Nannicini, 2011).

In practice, however, the degree to which democracies actually embody these ideals of representation, accountability, and competence varies a great deal. While low-income citizens have the right to vote, voter turnout is often far lower among these groups, potentially reducing the influence of this voting bloc on policy decisions that affect people's health (Reeves and Mackenbach, 2019). It is also not clear that electoral accountability is automatic: ‘it exists only if citizens connect specific policies to politicians and vote accordingly’ (Acharya et al., 2020). To take just one recent example, pandemic performance does not, on average, seem to have altered stated political preferences in a range of countries (Acharya et al., 2020; Neundorf and Pardos-Prado, 2022). Finally, candidate selection processes are not only competency competitions: attractiveness (Verhulst et al., 2010), name recognition (Cox and Katz, 1996), and party structures all play a sizeable role in both who can run and who wins elections (Hazan and Rahat, 2010).

One implication of this variation in the degree to which democracies live up to these theoretical ideas is that not all polities categorised as ‘democracies’ will have the same impact on health. This is because the degree to which democracies actually instantiate the mechanisms presupposed in the theories described above may vary a great deal and, as a consequence, the implications for health should vary too (Wang et al., 2018). We would anticipate, for example, that the health benefits of democratization would be largest in those contexts where marginalised and impoverished groups are represented, where political leaders are most accountable to the poor, and where selection processes are competitive and emphasise competence in the areas of coalition-building and administration (Acemoglu et al., 2010; Gerring et al., 2012).

To understand this variation, we need to look back to the creation of a country's democratic institutions. In these revolutionary moments, democratic movements seek to displace the existing order with a new set of institutions that create a path dependent set of structures that influence the shape of those democracies for years to come (Hite and Cesarini, 2004). This brings us back to the importance of the kind of movements (e.g., nonviolent versus violent) which create these revolutionary moments of democratization. Below we outline how the use of nonviolent tactics by democratic movements may produce different kinds of democracy and, by implication, how these different kinds of democracies could impact child mortality.

Nonviolent transitions appear more likely to create democratic institutions that represent deprived communities, make politicians accountable, and produce competent leaders who can build broad coalitions and actually implement their agendas. In part, this is because nonviolent transitions that succeed in delivering democracy are, by their nature, typically more consensual and accountable (Collier et al., 2008) than violent transitions, because to be successful they require broad support across a wider range of constituencies. Further, they are more likely to select competent leaders who can build coalitions and drive forward their agenda (Chenoweth and Stephan, 2011). In this sense, nonviolent resistance is successful precisely because it does the hard work of building a participatory movement that can forge coalitions in order to effect change (Kadivar et al., 2020).

The grassroots movements described above are usually only successful if they are able to generate ‘loyalty shifts’ that lead powerful constituencies, such as economic elites or military leaders, to break with those in power (Chenoweth and Stephan, 2011). In other words, elite groups realign themselves in response to pressure from below. This is precisely the kind of accountability mechanism that democracies are meant to foster in order to produce better health. In the Philippines, for example, the People Power Revolution of 1986, led by Cory Aquino, created a broad, nonviolent movement of approximately 2,000,000 people – students, workers, women's groups, and others – until eventually the media and the military publicly declared their support for the protestors (Mendoza, 2009). Movement building is difficult, especially under a repressive regime. The leaders of successful nonviolent movements have, by definition, already demonstrated their ability to build diverse movements that demand accountability from those in power. This kind of consensual, accountable democracy is more likely to pursue policies that improve child health, such as increased access to healthcare, including immunizations (Lake and Baum, 2001), clean water (Biser and Edwards, 2012), primary education (Harding, 2020), and public services more broadly (Deacon, 2009).

None of these features of nonviolent movements are true of violent movements, which are typically one of two kinds. The first is military coups and the second is small-scale movements driven by a very small constituency (typically young men) who do not need to build consensual coalitions in their transition to democracy (Chenoweth and Stephan, 2011), and whose often secretive organizations lack the same accountability mechanisms. The leaders of violent resistance are certainly competent in one important sense (they are successful) but this is not the same kind of competence that informs theories of democratization and health. Violent movements produce ‘specialists in violence rather than in politics’ (Licklider is cited in Chenoweth and Stephan, 2011) and it can be difficult to convince those that have been ‘killing people with considerable enthusiasm and success’ to not engage in more violence (Hartzell et al., 2001). Ecuador's democratic transition, for example, was the result of a bloody military coup. Elections did eventually occur but the military remained incredibly influential for at least a decade afterwards, refusing to address human rights abuses and keeping civil society actors weak (Isaacs, 1991). Violent transitions, therefore, produce a different kind of democracy than nonviolent transitions. In sum, our argument is similar to Huntingdon's assertion that: ‘governments created by moderation and compromise rule[d] by moderation and compromise. Governments produced by violence rule[d] by violence’ (1993: 207). In short, the kind of democracies produced by violent transitions are less likely to implement policies that reduce child mortality because they are less representative and accountable, and although the leaders may be competent, their competencies are in areas that are not linked to improving child health.

Our empirical contributions in this paper build on earlier work on the heterogeneous effects of democracy on health in two ways. First, we use an alternative measure of whether the transition to democracy is violent or not. Previous work used a measure of battle-related deaths, but the authors of that analysis found this was not correlated with their country-specific estimates of the effect of democratization on child mortality (Ramos et al., 2020). Instead, we categorise the violence of democratic transitions in terms of the nature of the resistance movement itself, rather than battle-related deaths post-transition. Second, we explore whether having a protest-led democratic transition constitutes a necessary cause of lower-than-expected child mortality following democratization. A cause (e.g., protest-led democratization) of an outcome (e.g., lower-than-expected child mortality post-transition) would be a *necessary* cause if observing the presence of the outcome necessarily implies the presence of that cause (Braumoeller and Goertz, 2000). This is a strong claim and our analysis goes beyond merely reporting correlations to assessing whether there is evidence for this necessary condition hypothesis.

## 2 Data and methods

To advance our understanding of these questions, we bring together data from a variety of different sources. Our dependent variable is the 55 country-specific point estimates of the long-run (more than 10-years) impact of democratization on child (under 5) mortality generated by Ramos et al. (2020) in their paper, and drawing on the estimates from the Institute for Health Metrics and Evaluation (IHME) (Rajaratnam et al., 2010). These are the best estimates available for under 5 mortality for countries that have gone though democratic transitions in the last 50 years. We base our models on the Ramos et al. estimates because they are the most recent and sophisticated long-run, country-specific estimates (and credible intervals) of the impact of democratization on child mortality. This is exactly the kind of estimates that we need to test our theory, which centres on how the type of democratic transition affects institution-building and norms in the long-term. On top of this, we conduct our own analysis of these democratic transitions, calculating new estimates of the impact of democratization on infant mortality. We then use these new estimates to observe whether our results are sensitive to using either the Ramos et al. estimates or these new estimates (see web appendix 1 for more details).

Ramos et al. (2020) focus on a set of countries that have all undergone a democratic transition and conduct an interrupted-time series analysis to estimate whether child mortality falls at the time of transition and whether the rate of change in child mortality is different post-transition compared to pre-transition. They estimate this model using Bayesian random slopes, allowing each country in their sample to have a different estimate. There are another 6 countries for which the democratic transition was too recent to be included in this part of their analysis. Their measure of democratic transitions comes from Cheibub et al.’s (2010) datasets, which code democracies between 1970 and 2009 according to the capacity for parties to lose elections.

Child mortality is an appropriate dependent variable because it is an important indicator of societal progress (Sen, 1998), a key component of population health (Reeves, 2021), and even a proxy for health inequalities, given that child mortality is often far higher among poorer households (Baker et al., 2018). We use Ramos et al.'s long-run (more than 10 years) estimates of child mortality because the true impact of democratization on health takes time to emerge (Gerring et al., 2012). We created two measures. For some of the analyses below, we coded ‘increases’ in child mortality as 1 if child mortality is lower-than-expected (i.e. compared to a counterfactual of no democratic transition) and the Bayesian credible intervals did not overlap with zero (and 0 otherwise). For other analyses we focus on decreases, coding as 1 countries where child mortality is lower-than-expected and the credible intervals did not overlap with zero (and 0 otherwise).

Our main independent variable is whether a country experienced a protest-led democratic transition or not, drawing on Chenoweth and Stephan's (2011) work on whether nonviolent resistance is more likely to lead to democratization. Their catalogue of transition types was produced through a systematic review of the democratization and conflict literatures, categorising major non-state resistance campaigns from 1900 to 2006, covering every country in the world. Protest-led transitions are coded as 1 (n = 33) and 0 otherwise (n = 18). We also create a measure of violence-led transitions, coding them as 1 (n = 14) and 0 otherwise (n = 37). We created this similar albeit distinct measure because some nonviolent transitions were not protest-led. For example, Sao Tome and Principe had an elite negotiated transition which might not produce the same kind of democracy post-transition as a protest-led transition. It is also worth noting that civil wars that precede a democratic transition would be classified as violent movements. There are 4 transitions in Ramos et al.'s paper that are not classified because we lack available data on the type of movement producing the transition (Bangladesh, Cyprus, El Salvador, and Sri Lanka), such that our dataset is made up of 51 cases in total (55 minus 4).

We also add covariates to this data (descriptive statistics and sources in Table 1). One set of covariates is concerned with characteristics of the country at the time of transition. These include: GDP per capita, adjusted for inflation and purchasing power, the percentage change in GDP (per capita, inflation adjusted, and in purchasing power parities) between the year of transition and five years later, and the level of child mortality per 1000 live births at the time of transition. Richer countries, countries with very fast post-transition growth, and countries with high child mortality may find it easier to reduce child mortality post-transition.

**Table 1:** Descriptive statistics for key variables and their sources.

Variable	N	Mean	Min	Max	Source
Country-specific estimate of democratization on child mortality	51	−0.01	−0.05	0.04	Ramos et al. (2020)
Protest-led transition (=1)	51	0.65	0	1	Chenoweth and Stephan (2011)
Violence-led transition (=1)	51	0.27	0	1	Chenoweth and Stephan (2011)
GDP per capita in year of transition	51	4610.91	302.50	19890.60	World Bank
Post-communist (=1)	51	0.35	0	1	Various sources
Proportion of years as a democracy since 1960	51	16.3	0	78.6	V-Dem
Child mortality rate at time of transition	51	68.79	7.78	247.17	World Bank
Change in GDP per capita between date of transition and 5 years after	50	5.64	−51.96	45.63	World Bank
Resource rents 5yrs after transition	51	3.73	0	17.60	World Bank
Net ODA (%GNI)	51	5.24	0	30.24	World Bank
Not colonised	22				Various sources
Colonised by North-Western European powers	14				Various sources
Colonised by Iberian powers	15				Various sources

A second set of covariates is concerned with whether the country becomes a ‘rentier’ state, relying on resource rents or aid to fund government activities and potentially becoming less responsive to citizens (Sandbakken, 2006). These measures include: total natural resources rents (% of GDP) 5 years after the transition and Net Official Development Assistance as a proportion of GNI.

A third set of covariates is concerned with other aspects of the democratic transition. These include: whether the country was formerly a communist country, the proportion of the years between 1960 and the year of transition that a country has been a democracy (Coppedge, 2018), and, finally, a measure of the coloniser country for the democratized country, because some colonisers were more extractive than others, which may also affect the kinds of democracy implemented in the present (Miller et al., 2018).

### 2.1 Analysis

Analytically, we proceed in six steps. First, we estimate bivariate OLS regression models that explore the association between the estimate of change in child mortality post-transition and our two measures of protest-led and violence-led democratic transitions.

Second, to explore the robustness of these correlations, we incorporate into our bivariate models the uncertainty around Ramos et al.'s estimates of the country-specific effects of democratization and child mortality. For example, child mortality is estimated to have increased by 0.043 deaths in Grenada but this could have been as low as 0.028 deaths or as high as 0.057 deaths per 1000 live births. The bivariate models mentioned in step 1 only use the point estimates and so those regression coefficients will not reflect the full range of likely values for child mortality post-transition (i.e., the upper and lower bounds of the Bayesian credible intervals). We therefore re-estimate the bivariate regression results 1000 times for each predictor (protest-led movement and violence-led movement) after adding a normally-distributed, country-specific error component to the dependent variable. This error component replicates the 95% credible intervals reported by Ramos et al. (2020) and therefore enables us to see whether our bivariate results would be incorrect under scenarios envisioned by the credible intervals.

Third, we estimate multivariate regression models which add the covariates mentioned above to the bivariate regression models. We only have a maximum of 51 observations and so adding covariates will very quickly reduce precision. Rather than select a particular set of potential confounders, we estimate 256 models, with each one representing a specific combination of the 8 aforementioned covariates. We then report what Young and Holsteen (2017) call the ‘robustness ratio’, a measure of how consistent the estimates are across these 256 models (determined by dividing the preferred estimate by the standard error of the estimates produced across all the models).

Fourth, we conduct a matching analysis using coarsened exact matching (Iacus et al., 2012). This matching technique increases the degree of similarity between countries experiencing different kinds of democratic transitions by producing an exhaustive set of categories that each capture a specific combination of characteristics found in the covariates (rich country with low corruption or rich country with high corruption). These categories are then used to identify exact matches and those cases that need to be removed from the analysis. More detail on the matching models can be found in Web Appendix 2.

In the fifth step of our analysis, we explore whether a protest-led (violence-led) transition is a necessary condition for lower (higher) than expected child mortality. In other words, we explore whether violent transitions *ever* lead to lower-than-expected child mortality and whether protest-led transitions *ever* lead to higher-than-expected child mortality. To do this, we use Braumoeller and Goertz's (2000) test for necessity under conditions of measurement error. We describe the logic of this test of necessary conditions as we walk through the results below (see also Web Appendix 3) but, in brief, this approach focuses on deviant cases (that is, cases which do not fit the hypothesis). Logically, any deviant cases would indicate that the condition is *not* necessary; but this is only true under conditions of zero measurement error, which is almost never true in the social sciences. Braumoeller and Goertz's approach to testing necessity asks: how likely is it that the presence of any deviant cases can be explained by measurement error? Braumoeller and Goertz argue that in situations in which (a) deviant cases are rare and (b) measurement error is likely, such as inaccuracies in separating ‘violent' and 'nonviolent’ campaigns, then it might be possible to establish a necessary condition despite the presence of deviant cases.

This test of necessary conditions assumes there is measurement error in the data and so in the sixth and final part of the analysis we explore that assumption using qualitative deviant case analysis. This method is, according to Seawright (2016), ideally suited to uncovering measurement error. We investigate the possibility of measurement error in any of the three main covariates: democratic transitions, child mortality, or the nature of the movement. We have four deviant cases: (1) two cases where child mortality rose after a protest-led transition (Argentina and Chile) and (2) two cases where child mortality fell after a violence-led democratic transitions (Romania and Sierra Leone). Here we explore whether there is any evidence of measurement error that would corroborate the conclusions drawn from the test of necessary conditions.

## 3 Results

### 3.1 Is the type of democratic transition correlated with change in the post-transition child mortality rates?

We begin our analysis by conducting a bivariate regression analysis (see Table 2) which shows that, on average, countries with a protest-led transition have lower-than-expected child mortality rates after the transition to democracy than countries with a movement that was violence-led (β = −0.017, *p* = 0.003). Violence-led transitions, meanwhile, have higher-than-expected child mortality rates after their transition on average (β = 0.020, *p* = 0.001). When we generate our own estimates of the effect of protest-led democratization on infant mortality post-transitions, we find a very high correlation between our estimates and Ramos et al.'s estimates. Moreover, we also find that transition type is correlated with infant mortality rates post-transition (see Appendix 1). Going beyond this, we explore the robustness of the results from the Ramos et al. data in three ways.

**Table 2:** Bivariate association between movement type and post-transition child mortality.

**Covariates**	Estimated deviation of child mortality rate from the pre-democratic transition trend	
	(1)	(2)
Protest-led transition (=1)	−0.017** (0.0055)	
Violence-led transition (=1)		0.020** (0.0058)
Constant	0.0047 (0.0044)	−0.012** (0.0030)
Observations	51	51
*R*^2^	0.17	0.20

Standard errors in parentheses. Protest-led and Violence-led transitions are distinct because not all nonviolent democratic transitions are necessarily protest-led.

*p < 0.05, **p < 0.01.

**Fig. 1:**
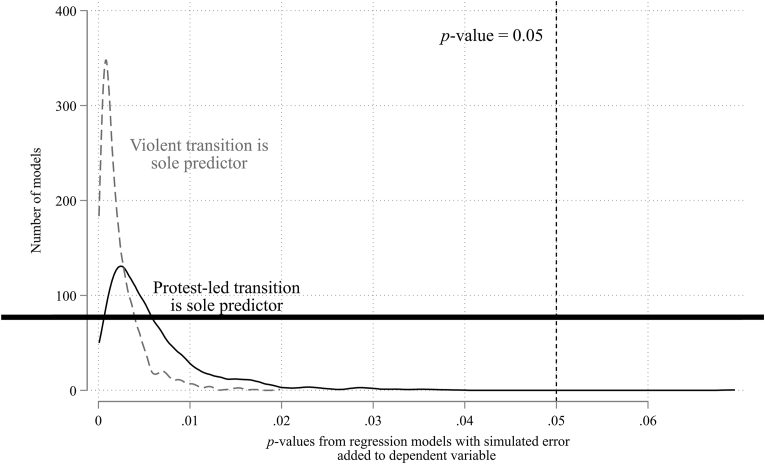
The association between movement type and post-transition mortality after simulating the error of the original estimates.

First, the estimates reported so far are based on the point estimates of the country-specific results reported by Ramos and colleagues. Do these estimates change if we model the uncertainty in their original estimates? Not only are all the coefficients in the same direction, but as shown in Fig. 1 (a kernel density plot of the *p*-values of these 2000 models – 1000 derived from using protest-led transitions as the predictor and another 1000 for violence-led transitions as the predictor), over 99.9% of these estimates for both outcomes have a p-value less than <0.05.

Second, we explore whether this association is altered after we adjust for all possible combinations of the covariates. Given the covariates we include and the number of observations we have available to us, we find a very high degree of robustness (Table 3). Both models meet Young and Holsteen's criteria of model robustness using the robustness ratio. Moreover, both models have a consistent direction and very high significance rates.

**Table 3:** Estimates of model robustness.

	Protest	Violent
Sign stability	100%	100%
Significance rate (*p* < 0.05)	100%	90%
Significance rate (*p* < 0.1)	100%	100%
Robustness ratio	−2.67	2.53
Possible control terms	8	8
Number of models	256	256

The third robustness test of the association between the type of democratic transition and child mortality is a matching analysis. The details of the matching and the results are described in Appendix 2 but our models suggest that even after matching on a key set of confounders (namely, GDP, child mortality at the time of transition, weak democratic transitions, corruption post-transition, and democratic history) we still find that protest-led transitions have lower-than-expected child mortality post-transition while violent transitions have higher-than-expected child mortality post-transition.

Viewed together, these results strongly suggest there is a robust correlation between the type of democratic transition that countries experience and whether that transition to democracy reduces child mortality post-transition.

### 3.2 Do violent transitions ever lead to lower-than-expected child mortality and do protest-led transitions ever lead to lower-than-expected child mortality?

Having established that protest-led transitions have lower post-transition mortality than what would be expected if they had not democratized, we now test two necessary condition hypotheses: 1) Is a transition that is *not protest-led* a necessary condition for higher-than-expected child mortality? and 2) Is a transition that is *not violent* a necessary condition for lower-than-expected child mortality? In Table 4, Table 5, we construct two tables to show which combinations of countries fit with or deviate from our expectations.

**Table 4:** Are there any cases of protest-led democratic transition that led to higher-than-expected post-transition mortality?

	Protest-led transition	Not protest-led transition
Not higher-than-expected mortality	**ALB, ARM, BEN, BOL, BRA, BGR, CZE, EST, GEO, GHA, GRC, HUN, IDN, KEN, LVA, LTU, MDG, MWI, MLI, MDA, MNG, NER, NGA, PHL, POL, SEN SER, SVK, SVN, UKR, URY**	CPV, ECU, GTM, HND, MEX, NIC, PRY, PER, PRT, ROM, STP, SLE, TUR
Higher-than-expected mortality	**ARG, CHL**	HRV, GRD, PAN, ESP, SUR

**Table 5:** Are there any cases of violence-led democratic transition that led to lower-than-expected post-transition mortality?

	Violence-led transition	Not violent-led transition
Not lower-than-expected mortality	**HRV, ECU, GRD, GTM, MEX, NIC, PAN, PRY, PER, PRT, ESP, SUR**	ARG, BEN, BOL, BRA, BGR, CPV, CHL, GEO, GHA, GRC, HND, IDN, KEN, MLI, NGA, SEN SER, SVK, SVN, TUR, UKR, URY
Lower-than-expected mortality	**ROM, SLE**	ALB, ARM, CZE, EST, HUN, LVA, LTU, MDG, MWI, MDA, MNG, NER, PHL, POL, STP

We focus on whether there are deviant cases in protest-led transitions (Table 4) or violence-led transitions (Table 5). Our data contains 33 cases of protest-led democratic transitions. Only two of these show any clear evidence of higher-than-expected child mortality in the years after this democratic transition (Table 4). These cases could be “true” deviant cases, invalidating the hypothesis that a transition that is not protest-led is a necessary condition for higher-than expected child mortality (since lower-than-expected child mortality can follow protest-led transitions in these two cases). Or, they could be explained by measurement error in child mortality, type of transition, or timing of democratic transition. As discussed above, Braumoeller and Goertz provide a method for evaluating the likelihood that these deviant cases are explained by measurement error.

We want to evaluate whether a transition that is *not* protest-led is a necessary condition for higher-than-expected child mortality. There are 2 deviant cases in Table 4 (Argentina and Chile) and the total sample is 33 observations. Deviant cases are, therefore, 6.06% of all observations. We first calculate the lower bound of the proportion of deviant cases in this sample using a one-sided 95% confidence interval from a binomial distribution, which is 0.0109 (or 1.09%). This implies that the population proportion of deviant cases is very likely to be greater than 1.09%.

The p_I_-test of necessity states that we ‘reject the hypothesis of necessity’ (i.e. the hypothesis that protest-led transitions are a necessary condition for lower child mortality rates post-democratic transition) ‘if the lower bound of the one-sided 95 percent confidence interval around $\hat{p}$ (which is the sample proportion of the cases in that cell) is greater than the estimated error rate’. In our data, $\hat{p}$ is 6.06% and the one-sided confidence interval for this proportion is 1.09%. To reject Braumoeller and Goertz's p_I_-test of necessity, we would need to observe more deviant cases in our data than could be accounted for by measurement error. We do not have an accurate estimate of the measurement error in the classification of the democratization movements and so we rely on previous evidence in this area. For Braumoeller and Goertz, anything less than 2% is an ‘enviably low error rate’ (<2%). In our data, we would need to have a very high degree of confidence that there is *no* measurement error in our data (less than 1% of cases would have to be misclassified) for us to conclude that our deviant cases are “true” counter examples and that protest-led transitions are not a necessary condition for healthy democratic transitions. In sum, we find evidence that protest-led transitions could be a necessary cause of lower-than-expected child mortality, despite the existence of two deviant cases, because these deviant cases could reasonably be due to measurement error.

Next, we consider the same set of tests for whether violence-led democratic transitions ever reduce child mortality (Table 5). This allows us to test whether a transition that is *not* violent is a necessary condition for lower-than-expected child mortality. There are 2 deviant cases in Table 5 (Romania and Sierra Leone) and the total sample is 14 observations. Deviant cases are 14.29% of all observations. We again calculate the lower bound of the proportion of deviant cases in this sample using a one-sided 95% confidence interval from a binomial distribution, which is 0.026 (or 2.6%). This implies that the population proportion of deviant cases is very likely to be greater than 2.6%. Again, this is a very low error rate (<3%), implying that less than 3% of deviant cases would have to be measurement error in order for us to reject the hypothesis of necessity (i.e. the hypothesis that violent-led transitions are a necessary condition for democratic transitions to lead to lower-than-expected child mortality). Since measurement error is likely to exceed this threshold, we cannot reject the hypothesis of necessity for violent-led transitions.

Viewed together, these tests provide some evidence in favour of our hypotheses, namely (1) that protest-led democratic transitions never lead to higher-than-expected child mortality rates and (2) that violence-led democratic transitions never lead to lower-than-expected child mortality rates.

### 3.3 Exploring our deviant cases

However, our data does contain some deviant cases. These cases are useful in both (i) uncovering measurement error and (ii) potentially also uncovering other sources of causal heterogeneity. Here we focus on the four deviant cases that inform the analyses above: (1) the two cases where child mortality rose after a protest-led transition and (2) the two cases where child mortality fell after a violence-led democratic transitions.

### 3.4 Rising child mortality following a protest-led democratic transition: Argentina and Chile

While there is almost certainly measurement error in the dependent variable for all of our data, the IHME estimates of child mortality for Argentina and Chile are notable because these mortality estimates were based on two data sources, the census and the vital registration system, which diverged significantly from each other (Ramos et al., 2020). While the census data results in considerably lower mortality estimates in the early periods, IHME's Bayesian inference model follows the vital registration system data most closely despite the fact, as they acknowledge, that this data source is very likely inaccurate. This is unlike some of the other countries in our data, such as Armenia, Benin, Brazil and others, where the various data sources underpinning estimates of child mortality are more consistent and where the modelled estimate closely follows the census data estimate. On top of this, the transitions to democracy in both Argentina and Chile occurred when their child mortality rates were already relatively low compared to the other countries in our analysis (less than the median in this sample). Thus, the notion that mortality was lower-than-expected post-transition might be partly driven by the natural slowdown in child mortality that results from approaching the lower bound. Indeed, in the 1980s both countries see improvements in child mortality decelerate once they fall below 40 deaths per 1000 births.

On top of this, while both countries experienced strong, protest-led democratic transitions, both countries also went through profound economic changes during this period. Argentina witnessed hyperinflation and implemented a program of massive economic liberalisation post-transition (Levitsky, 2005), producing higher income inequality, falling real wages among low-income households, and rising unemployment (Cruces and Gasparini, 2008). This period of intense economic dislocation almost certainly led to increased deaths, like later Argentinian crises in the 1990s (Cruces et al., 2012), although there is debate about the effect of the reforms in this period (Galiani et al., 2005). While Chile did not experience the same economic woes as Argentina, it did continue to pursue economic liberalisation policies initiated under Pinochet. This was partly because the same elite remained in power, which itself raises questions about the degree to which this was a genuine democratic transition (Oppenheim, 2007). Indeed, the slow-down in Chile's progress on child mortality began shortly after these reforms in the early 1980s, before the transition to democracy occurred.

In both instances, therefore, measurement error, low child mortality rates at the point of transition, and economic liberalisation may help us explain why these protest-led transitions to democracy subsequently had higher-than-expected mortality.

### 3.5 Falling child mortality following a violence-led democratic transition: Romania and Sierra Leone

We also observe some violence-led democratic transitions that see falling rates of child mortality post-transition. Romania is one of these countries and it is, in many ways, an unclear case in our analysis. Perhaps the most salient issue related to measurement error is whether it experienced a violence-led transition at all. While Chenoweth and Stephan (2011) classify the 1989 rebellion that eventually overthrew Ceausescu as a violent campaign, this rebellion was preceded by nonviolent attempts to remove him from power. These proved unsuccessful and were violently suppressed. This led to some violence in response but even this was mostly unarmed, leading some to argue it was not an *armed* violent transition (Kadivar and Ketchley, 2018). Even the “violent” December 1989 rebellion, at the end of which Ceausescu and his wife were executed after a trial on Christmas day, included largely nonviolent protests. For example, during Ceausescu's now famous, nationally televised live speech on 21 December, his power was directly challenged by a supposedly spontaneously-formed crowd of ‘supporters’ (Siani-Davies, 2005). Ironically, Ceausescu had created a crowd of protestors and this unarmed group of nascent revolutionaries were then attacked by government forces. On top of this, Romania is unusual because it is not entirely clear that it should be in the dataset at all – some scholars argue that it did not ‘fully transition to a democracy’ because of the high levels of corruption that continue to plague the country (Chenoweth and Stephan, 2011). Another potentially important factor is that Romania's democratic transition ran parallel to its economic transition from a communist to a capitalist system. Many of the post-communist countries in our data (11 out of 18) experienced a significant reduction in child mortality post-transition, and this is a factor that comes out of Ramos and colleagues' (2020) earlier work as a possible explanatory factor behind the patterns they observe.

Sierra Leone is our next deviant case, another country in which child mortality rates seemed to rise after a protest-led transition. Here, again, it is a complex case. Ramos and colleagues identify Sierra Leone's democratic transition as occurring in 1998, but this is slightly earlier than other datasets. For example, the Varieties of Democracy (Coppedge, 2018) data recognise Sierra Leone as democracy from 2003 onwards, which suggests there may be some measurement error in the transition date. This could affect the accuracy of the long-term effect estimate from Ramos and colleagues. Perhaps most importantly, however, Sierra Leone was wracked by a civil war prior to the democratic transition, and, if we take 1998 as the date of transition, the civil war continued after it too. It was only in January 2002 that President Kabbah declared the eleven-year-long Sierra Leone Civil War officially over. Sierra Leone's first elections then, held in 1996, ‘took place within the context of an ongoing civil war’ (Wai, 2015: 219). These were not just elections, they were ‘a conflict resolution strategy’ (Wai, 2015). In this respect, the armed conflict was more than just a violent insurrection, it was a ‘civil war’ which brought about huge loss of life; by some estimates at least 50,000 people were killed in the conflict between 1991 and 2002 (Bellows and Miguel, 2009). The reduction in mortality that occurred in the years following 1998 is therefore perhaps best explained by the slowdown in the violence during this civil war rather than the transition to democracy, which did not fully occur until 2003.

Both Sierra Leone and Romania are cases in which measurement error could plausibly explain their deviation from expectations. Our deviant case analysis, therefore, has confirmed the intuition behind our tests of necessary causation, namely that measurement error potentially exists within those cases which appear to contradict the primary expectation of our theory. Even if we assume that these cases are measured correctly, our deviant case analyses potentially suggest other variables that may explain why democratic transitions can sometimes lead to lower-than-expected child mortality, including large-scale economic liberalisation. Future work should take up these questions.

## 4 Discussion

In this paper, we have grappled with the question of why democratization seems to improves health in some contexts but not others. We have argued that the type of movement that generates the democratic transition may explain this variation. Our data reveal a surprisingly stable association between nonviolent transitions and lower-than-expected child mortality after the transition. We find evidence that violence-led transitions almost never lead to lower-than-expected child mortality post-transition and that protest-led transitions almost never lead to higher-than-expected mortality post-transition. In both analyses, we find some evidence that protest-led transitions may even be a necessary condition for avoiding increased mortality.

It is important to be clear about exactly what this claim entails. This does not mean that protest-led transitions are a sufficient condition (Braumoeller and Goertz, 2000), i.e. that they *always* lead to lower-than-expected child mortality post-transition. They do not. In the set of democratic transitions in this data, ∼60% of protest-led transitions have a credible interval which crosses zero and so protest-led transitions do not *always* lead to lower-than-expected child mortality post-transition. But, it is also true that the vast majority of protest-led transitions (>80%) have, according to Ramos and colleagues’ models, a point estimate below zero (even if the credible intervals for some of these cross zero). What our data do suggest is that protest-led transitions almost *never* lead to higher-than-expected child mortality.

Despite focussing on child mortality, these findings have implications for our understanding of the link between democracy and health more broadly. Most explanations of this relationship rest on the assumption that governments will implement policies that ensure the health and longevity of their citizens’ children (Harding, 2020; Wang et al., 2018). Our data suggest that the degree to which this occurs in the 10 years following this transition is partially predicated on how the transition to democracy occurs. This is because a violent transition is culturally and institutionally different from a nonviolent one (Bethke and Pinckney, 2021). The participants in nonviolent movements are more likely to codify the emerging norms of nonviolent contestation into democratic institutions and it is this codification which allows the kind of electoral competition and negotiation that produces egalitarian policies in our models of political economy (Chenoweth and Stephan, 2011). Nonviolent insurgency is more likely to lead to a stable and more inclusive democracy and to civil peace after the reform (Bethke and Pinckney, 2021; Ives, 2021), which is likely to further decrease child mortality. Conversely, violent conflict will produce different kinds of political competition that are not predicated on democratic competence or even value-fit. While conflict may produce greater social cooperation, this greater solidarity is experienced largely among the in-group (Bauer et al., 2016) and may therefore lead to greater polarization. Moreover, the fear of popular discontent in response to such violence may make regimes less likely to institutionalise the kind of democratic values and ideals in the period after the reform that are assumed to produce better health.

There are, of course, important limitations to our analysis. The first is that we did not have data on the type of movement leading to a democratic transition for every transition in Ramos and colleague's data. It is, however, unlikely that these few missing cases would substantially alter our regression results, although they could be more challenging for our test of necessity if they turn out to be deviant cases. Second, lower-than-expected child mortality post-democratic transition is likely an outcome with more than one causal chain and so while our argument is that movement type matters this does not mean that movement type is the only factor that matters. There may be other factors (or, more likely, combinations of factors) that will explain lower-than-expected mortality after transitions to democracy. Third, we cannot entirely rule out the possibility that violent transitions have higher-than-expected mortality because such transitions lead to large conflicts, such as civil wars, after the transition to democracy (Ghobarah et al., 2003). This is unlikely, however, because as Ramos et al. observe, there is no correlation between the size of conflicts post-transition and child mortality rates over the long-run. Plus, among the 10 countries with conflict post-transitions, only Croatia saw lower-than-expected mortality. Most of the countries that did see conflict post-transition did not see child mortality increase and therefore post-transition conflict does not seem to be a driver of increased child mortality after democratization. Fourth, it is plausible that some of cases that appear to be consistent with our hypotheses may also be due to measurement error. Fifth, other aspects of conflict are not coded in our analysis. For example, violent but unarmed protestors can have a positive impact for democratic transitions (Kadivar and Ketchley, 2018) and future work should consider whether such movements are more or less likely to contribute to health improvements post-transition. While our work looks at long-run child mortality, it is still only a relatively short time (decades not centuries), and more work will be needed to discover whether even these violent transitions eventually bring significant improvements in child mortality. Sixth, child mortality is different in important ways from other health outcomes and so these findings may not apply to other health outcomes. We hope future work takes up these questions.

Our paper contributes to the growing body of work exploring the political economy of health (Reeves, 2021), and confirms that political institutions affect the health of populations (Wigley and Akkoyunlu-Wigley, 2017). We cannot simply assume, however, that democratization always improves child mortality nor population health more broadly. This is partly because some health crises might prove challenging for democracies but also because not all forms of democracy actually embody the ideals of representation, accountability, and competence that underpin the theoretical explanations of the relationship between democracy and health. Moreover, our results also stress the path dependent nature of these institutions, i.e. the connection between the type of democratization movement and the kind of democracy post-transition. This durability means that institutionalising democratic ideals, even within nominally democratic countries, will be far from easy. Remaking political institutions cannot happen overnight, of course, and some ideals are hard to reach given an existing set of institutional arrangements. And yet, there has been a recent wave of democratic backsliding – which has involved institutional reforms moving away from these ideals. What is particularly striking about these anti-democratic reforms is that they seem to have already caused negative health consequences (Wigley et al., 2020). The potential health consequences of this wave of authoritarian pressures requires even more careful reflection on how exactly deepening democracy may ensure good health for all.

## Authorship statement

**Aaron Reeves**: Conceptualization, Methodology, Software, Formal analysis, Investigation, Writing – original draft. **Laura Sochas**: Conceptualization, Writing – review & editing.

## Funding

AR and LS acknowledge funding from the 10.13039/100010269Wellcome Trust (220206/Z/20/Z).

## Data Availability

Data and code is available here: https://github.com/asreeves/democracy-non-violence
