# Supplementary material for: When do democratic transitions reduce or increase child mortality? Exploring the role of non-violent resistance
Source: Soc Sci Med. 2022 Dec;314:115459. doi: 10.1016/j.socscimed.2022.115459 (PMC10926273; doi:10.1016/j.socscimed.2022.115459)
Supplement: Multimedia component 1 [file mmc1.docx]

Web Appendix 1: Re-estimating country-specific impacts of democratic transitions on infant mortality

While we believe Ramos and colleagues’ estimates are likely the best and most comprehensive estimates of the country-specific effect of democratization on under 5 mortality, we want to ensure that our analyses are consistent with alternative ways of modelling these data. We therefore have three main objectives in this web appendix. First, we create our own estimates of the country-specific effect of democratization on under 5 mortality, using a less sophisticated but similar approach to Ramos and colleagues. Second, we use these newly created estimates to check the robustness of the regression results reported in the paper. Third, we explore whether our hypotheses are confirmed using alternative but more familiar regression models (such as linear regression and panel regression models with country fixed-effects).

We use the same data as Ramos and colleagues. Under 5 mortality rates come from an updated version of Rajaratnam et al (2010) which is part of the work carried out by the Institute for Health Metrics and Evaluation (IHME). The underlying data are drawn from vital registration systems, summary birth histories, and complete birth histories, and were largely collected by independent international agencies; the data were then combined using Gaussian process regression, which captures the uncertainty caused by sampling and non-sampling error across data types. We also use the same classification of democratic transitions as Ramos et al (originally from Cheibub et al (2010)).

1. We start by replicating Ramos’ estimates:

Ramos and colleagues use an interrupted time series design to estimate the effect of democratic transitions on child mortality. Their Bayesian random effects model (with country-specific random intercepts and random slopes) estimates under 5 mortality in terms of a log-linear time trend, a binary variable indicating whether the transition to democracy has occurred, and an interaction between these variables. They use this model to estimate the counterfactual trends for child mortality in the absence of a democratic transition.

We implement a simpler model – key differences include: (a) We do not distinguish between the immediate change in levels at the time of the democratic transition and the change in the trend. (b) We do not use random effects. (c) We only use data pre-democratic transition instead of all data (pre- and post-transition). (d) We estimate the model using frequentist rather than Bayesian models.

Our simpler model estimates counterfactual trends in under-5 mortality using fixed/non-random country-specific time trends pre-transition:

(1) Under5_it_ = βcountry_1i_ + θyear_t_ + δcountry × year_i,t_ + ε_i_

Where *Under5* is the under 5 mortality (on a log scale) for country i at time t; *country* is a vector of binary variables for each country included in the model (β is the vector of associated coefficients), *year* is a linear time trend, and the interaction between country and year estimates a country-specific time trend for each country (δ is the vector of coefficients for these country-specific interaction terms). We then use this model to predict the counterfactual of what would have happened to under 5 mortality in the absence of the transition to democracy.

In web figure 1a, we show the data for Mongolia. The reddish line is the IHME estimated trend in under 5 mortality in Mongolia between 1970 and 2010. The orange line is our estimate of the counterfactual. It is based on under 5 mortality before 1990 because Mongolia transitioned to a democracy in 1990. The two lines fit together very tightly before 1990 and afterwards they start to diverge. The difference between these two lines is our estimate of whether under 5 mortality was lower-than-expected or higher-than-expected. In this instance, Mongolia saw lower-than-expected under 5 mortality after its democratization.

Web Figure 1b: Observed and counterfactual trend in under 5 mortality in the Czech Republic.


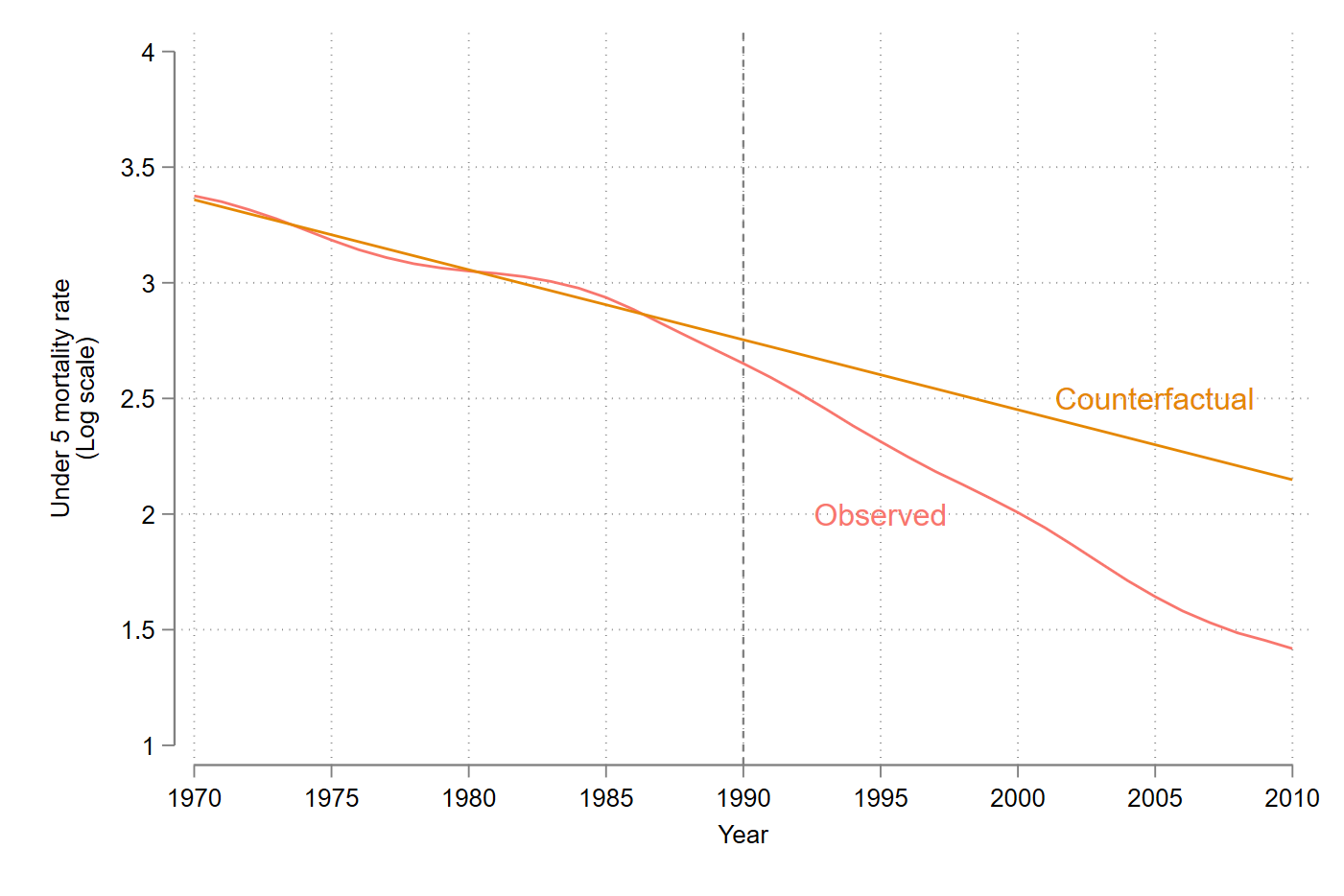


We calculate the difference between the observed and the counterfactual for all of our countries 10 years after they transition to a democracy. We then compare them to the estimates from Ramos and colleagues, finding they are very similar. Indeed, the correlation between our estimates and the estimates from Ramos et al is 0.913 (see Web Figure 1b).

Web Figure 1b: Bivariate association between our estimates of the long-run impact of movement type and post-transition child mortality and the estimates from Ramos


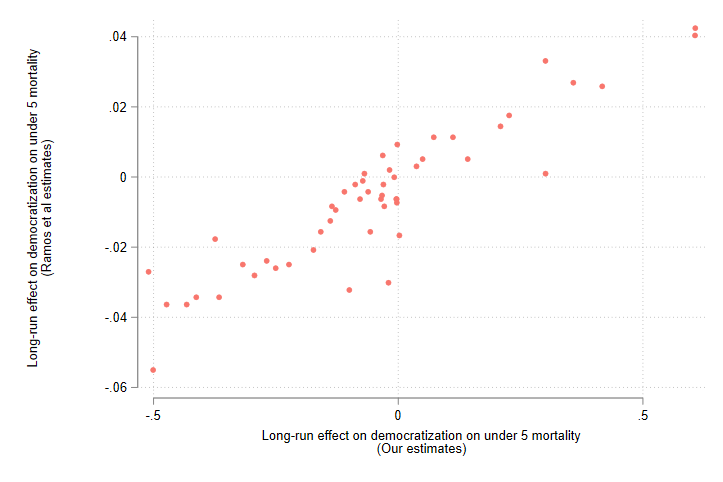


1. Next, we test whether our main paper’s regression results hold, using the same 51 countries who experienced a democratic transition and the same measures of protest-led and violence-led transitions that are used in the main text of our paper.

This is effectively the same procedure used by Ramos and colleagues in Part 6 of their paper. The results are consistent with each other. We standardized the coefficients and find that they are comparable for both sets of estimates.

Web Table 1a: Bivariate association between movement type and post-transition child mortality

|  | **Estimated deviation of child mortality rate from the pre-democratic transition trend** | | | |
| --- | --- | --- | --- | --- |
|  | Main paper (based on Ramos et al estimates) | Appendix  (based on our estimates) | Main paper (based on Ramos et al estimates) | Appendix  (based on our estimates) |
| **Covariates** | (1) | (2) | (3) | (4) |
| Protest-led transition (=1) | -0.41**  (*p* = 0.003) | -0.33*  (*p* = 0.018) |  |  |
|  |  |  |  |  |
| Violence-led transition (=1) |  |  | 0.45**  (*p* = 0.001) | 0.38**  (*p* = 0.006) |
|  |  |  |  |  |
| Constant | 0.0047 (0.0044) | 0.060 (0.056) | -0.012^**^ (0.0030) | -0.11** (0.038) |
| Observations | 51 | 51 | 51 | 51 |
| *R*^2^ | 0.17 | 0.11 | 0.20 | 0.14 |

P-values in parentheses. Protest-led and Violence-led transitions are distinct because not all non-violent democratic transitions are necessarily protest-led.

^*^ *p* < 0.05, ^**^ *p* < 0.01

1. Finally, we want to establish whether our findings are still valid if we analyse the type of democratic transition relative to a control group of countries that did not democratise.

We can use these new estimates to explore the impact of protest-led and non-protest led transitions to democracy, compared to permanent autocracies. Ramos et al do not explore their data in this way.

We start with a simple model which uses permanent autocracies as the control group (or baseline) and then compares this set of autocracies to countries that (1) have had a violent transition to democracy and (2) those that have had a protest-led transition to democracy. We estimate a linear regression model of the form:

(2) DiffUnder5_it_ = **β**Dem_it_ + θyear_t_ + **δ**Dem × year_it_ + ε_i_

Where *DiffUnder5* is the difference between the observed (on a log scale) and the counterfactual under 5 mortality rates for country i at time t. If *DiffUnder5* is negative, it means that under 5 mortality is lower-than-expected whereas if *DiffUnder5* is positive, then under 5 mortality is higher-than-expected. *Dem* is a 3-level categorical variable in which 0 = countries that are permanent autocracies, 1 = countries that have seen a non-protest-led transition to democracy and 2 = countries that have seen a protest-led transition to democracy (**β** is the vector of coefficients estimating the difference between these types of democratization and permanent autocracies). *year* is a linear time trend, and the interaction between the type of democratic transition (*Dem*) and *year* estimates the change in the slope in the difference between the observed and counterfactual trend in Under 5 mortality (**δ** is the vector of associated coefficients). Standard errors are clustered to adjust for repeated observations over time.

To make this more concrete, we have graphed the difference between the observed and the counterfactual under 5 mortality rates for both Mongolia and Chile. We have chosen these two countries primarily because they both transitioned to democracy in 1990, making the graph simpler to read, and because Mongolia had a protest-led transition to democracy whereas Chile had a violence-led transition. Our model, in effect, calculates the difference between Chile and Mongolia prior to 1990 (this is the β and the θ in the model above). In these two countries, there is almost no difference in the levels or the slopes between these two countries. Then, we estimate any change in the slopes between Chile and Mongolia after 1990 (this is δ in the equation above). As can be see, Chile and Mongolia move along different paths, with Chile witnessing higher-than-expected mortality while Mongolia observes lower-than-expected mortality.

Web figure 1c: The difference between the observed and the counterfactual Under 5 mortality rate for Panama and the Czech Republic before and after the transition to democracy


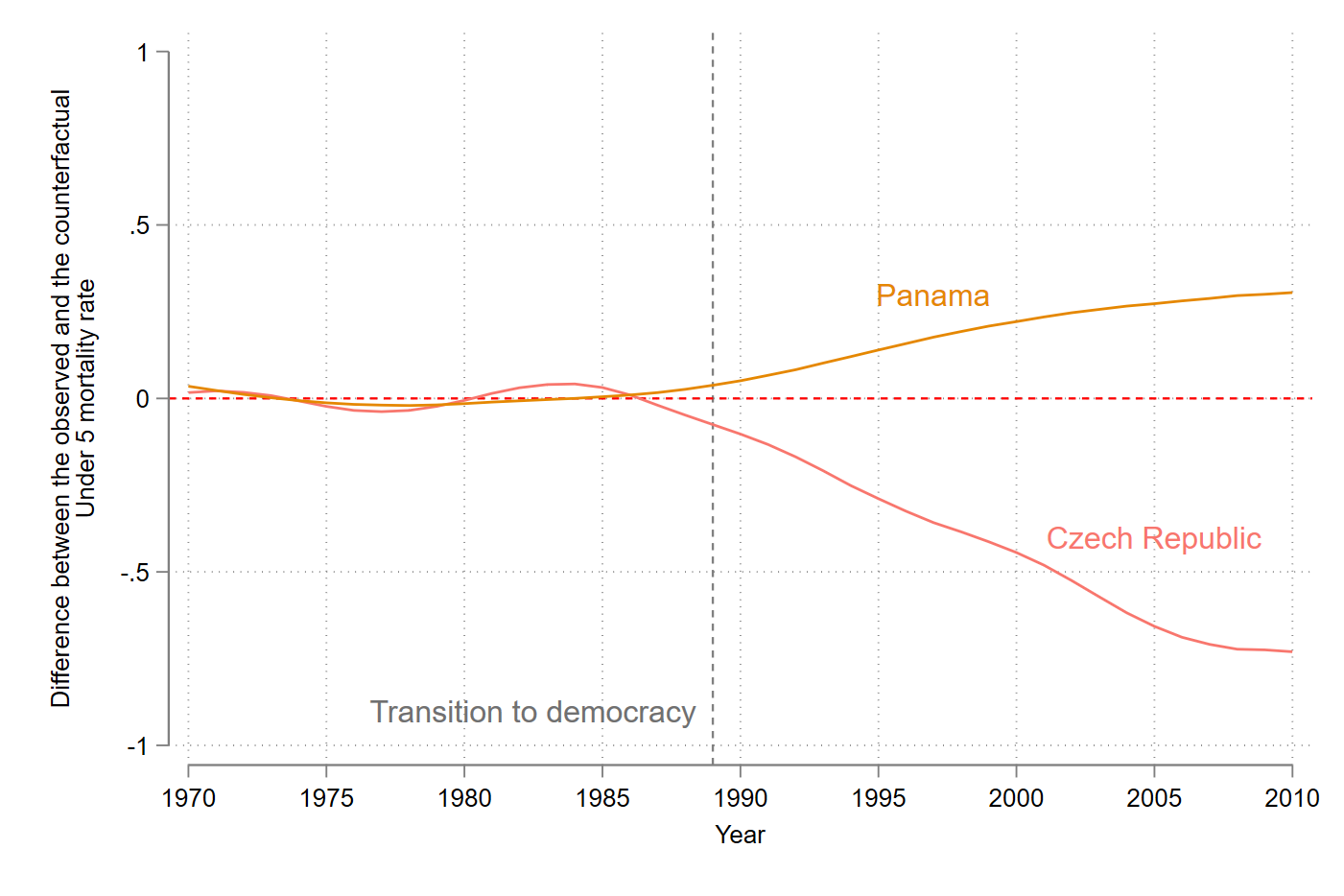


In fact, these two countries are good exemplars of the wider pattern. We find that, on average, our non-protest-led transitions have (like Panama) higher-than-expected under 5 mortality compared to permanent autocracies, although the p-value is greater than 0.05 (.0050, 95% CI: -.0023 to .013). We also find that, like the Czech Republic, protest-led transitions have lower-than-expected under 5 mortality compared to permanent autocracies (-.0066, 95% CI: -.00092 to -.012). When we directly compare protest-led and non-protest-led transitions, we find that protest-led transitions have lower-than-expected under 5 mortality compared to non-protest-led transitions (*p* = 0.017).

Web Table 1b: Exploring whether movement type is associated with higher- or lower-than-expected under 5 mortality post-transition using panel regression models

|  | Difference between observed and counterfactual under 5 mortality rate  (Panel regression) | Main paper (based on Ramos et al estimates – non-standardized) |
| --- | --- | --- |
| Variables | (1) |  |
| Non-protest-led transition (compared to autocracies) | 0.0047  (*p* = 0.237) | __ |
| Protest-led transition (compared to autocracies) | -0.0072  (*p* = 0.009) | __ |
| Protest-led transition (compared to non-protest-led transitions) | -0.012  (*p* = 0.017) | -0.017  (*p* = 0.003) |
|  |  |  |
| Country-years | 4348 | 51 |
| Countries | 128 | 51 |

Finally, as a sensitivity test, we estimate time series cross-sectional regression models with the same dependent variable but now we add country-specific fixed effects, which account for country-specific differences that are stable over time. The model we estimate is:

(3) DiffUnder5_it_ = βDem_it_ + θyear_t_ + **δ**Dem × year_it_ + γcountry_i_ + ε_i_

Where *DiffUnder5* is the difference between the observed (on a log scale) and the counterfactual under 5 mortality rates for country i at time t. If *DiffUnder5* is negative it means that under 5 mortality is lower-than-expected whereas if DiffUnder5 is positive then under 5 mortality is higher-than-expected. *Dem* is a 3-level categorical variable in which 0 = countries that are permanent autocracies, 1 = countries that have seen a nonprotest-led transition to democracy and 2 = countries that have seen a protest-led transition to democracy (β is the vector of coefficients estimating the difference between these types of democratization and permanent autocracies). *year* is a linear time trend, and *country* is a series of dummy variables for each country (i.e.: the country fixed effects). Standard errors are clustered to adjust for repeated observations over time.

The coefficients of interest is **δ** and this estimates the difference in the country-specific slopes before and after the transition to democracy, depending on the type of transition.

Web Table 1c: Estimates of the trend in under 5 mortality (relative to the estimated counterfactual) for permanent autocracies, violent transitions to democracy, and protest-led transitions to democracy using panel regression models

|  | Trend in the difference between observed and counterfactual under 5 mortality rate  (Panel regression) |
| --- | --- |
| Variables | (1) |
| Permanent autocracies | -0.0004  (*p* = 0.553) |
| Non-protest-led transition | 0.0093  (*p* = 0.097) |
| Protest-led transition | -0.013  (*p* = 0.004) |
|  |  |
| Protest-led transition (compared to non-protest-led transitions) | -0.023  (*p* = 0.002) |
|  |  |
| Country-years | 5077 |
| Countries | 128 |

We find that protest-led transitions to democracy have lower-than-expected under 5 mortality than countries (-0.013, 95% CI: -0.0042 to -0.022) and that this is different from those countries that did not have a protest-led transition to democracy (-0.023, 95% CI: -0.001 to -0.021).

In sum, after re-calculating Ramos and colleagues’ estimates and exploring the relationship between movement type and under-5 mortality in the same publicly available data used by Ramos et al, we still find that protest-led transitions to democracy are associated with lower-than-expected under 5 mortality whereas non-protest-led transitions to democracy are not.

Web Appendix 2: Description of the matching procedure

Coarsened Exact Matching (CEM) is a partial matching procedure. We match on the following variables: GDP at the time of transition, the level of child mortality at the time of transition, whether it was a weak democratic transition or not, the level of corruption post-transition, and democratic history. Each of these are either empirically important based on earlier work or theoretically important variables.

CEM splits all variables into bins or categories. In some cases, we allow these bins or categories to reflect the pre-defined categories of all the variables included in the model (e.g., whether it was a weak democratic transition or not). In other cases, we allow the model to select cut points within the variable. We split GDP and corruption into quartiles and child mortality into two categories. All the other variables are binary.

Adding all these variables together creates 21 different possible combinations (or strata) and the CEM algorithm seeks to match the observations that went through a protest-led transition to other cases in the data (we use the same procedure for matching the violence-led transitions). Only 6 strata have matched countries. It is possible to have more than one match in each strata and so the matching is weighted to reflect the uneven distribution of the data across these strata. CEM is usually assessed using a global fit statistic ζ_1_ (or *L_1_*). This fit statistic tells us how imbalanced the data sets are before the matching procedure (1 = completely separable or no-overlap while 0 = perfectly balanced). A data set in which the data set is completely separable would imply that the treated units (e.g., protest-led countries) are entirely different on all the covariates from the untreated units (e.g., not protest-led countries). A balanced data set is one in which the treated and control units overlap completely so that countries with protest-led democratic transitions are similar to countries whose democratic transitions were not protest-led in terms of matching variables (listed above).

In our analysis, before the matching procedure for the protest-led model, ζ_1_ is 0.731. This suggests that protest-led countries are quite different from countries that not protest-led in terms of the matching variables. After the matching procedure, the ζ_1_ has fallen to 0.188, which we regard as a significant improvement. For the violence-led model, ζ_1_ is 0.734 while after the matching procedure ζ_1_ has fallen to 0.313. The matching is not perfect, of course, but CEM is by definition an improvement over the imbalance observed in the raw data.

In the table below we report results from 6 models across 2 predictors. Models 1-3 focus on protest-led transitions and models 4-6 focus on violence-led transitions. In columns 1 and 4 we report the results from the original OLS regression models for both of our main predictors. Columns 2 and 5 show the results once the data has been matched and weighted, hence the sample size is substantially reduced. Columns 3 and 6 report results from the ‘doubly robust’ version of the matched and weighted sample, that is, with the covariates used in the matching analysis added to the regression model on their original scales to capture any residual variation across the covariates.

Table: Association between movement and post-transition mortality using a matching procedure

|  | **Estimated deviation of mortality rate from the pre-democratic transition trend** | | | | | |
| --- | --- | --- | --- | --- | --- | --- |
|  | (1) | (2) | (3) | (4) | (5) | (6) |
| Protest-led transition (=1) | -0.017^**^  (0.0055) | -0.029^**^ (0.0084) | -0.023^**^ (0.0076) |  |  |  |
|  |  |  |  |  |  |  |
| Violence-led transition (=1) |  |  |  | 0.020^*^  (0.0057) | 0.021^*^ (0.0085) | 0.019^*^ (0.0078) |
|  |  |  |  |  |  |  |
| Doubly robust model | N | N | Y | N | N | Y |
|  |  |  |  |  |  |  |
| Observations | 51 | 25 | 25 | 51 | 25 | 25 |
| *R*^2^ | 0.17 | 0.34 | 0.66 | 0.20 | 0.21 | 0.59 |

*Notes:* Standard errors in parentheses. We estimate doubly robust matching models (i.e., adding the covariates we used in the matching process to the regression model too) using the following covariates: GDP at the time of transition, the level of child mortality at the time of transition, whether it was a weak democratic transition or not, the level of corruption post-transition, and democratic history.

Web Appendix 3: Description of the tests of necessity

Here we provide a more detailed description of the necessary conditions tests. To illustrate this, we focus on one of our key hypotheses:

Non-protest led transitions are a necessary condition for high-than-expected child mortality post-transition.

One implication of this hypothesis of a necessary condition is that the proportion of cases which *do not* experience a violence-led democratic transition but which see child mortality *increase* post-transition should be zero. This is because higher than expected child mortality post-transition should be impossible if a non-protest led transition does not occur. To explore this, we construct a truth table. Our tables show us that there are some cases which experience a protest-led democratic transition but which also saw child mortality increase post-transition. These are deviant cases and potentially are evidence that non-protest led transitions are not a necessary condition for higher than expected child mortality post-transition.

However, whether these deviant cases are indeed evidence against necessity is dependent on how confident we are that there is zero measurement error in our data. There is almost always measurement error and so we need to explore how much measurement error there is relative to the number of counterexamples against the hypothesis. A test of necessity would therefore be, according to Braumoeller and Goertz, to reject the necessary condition hypothesis if the estimated error rate of the data is significantly lower than the sample proportion of deviant cases.

Consider a scenario in which there are 33 cases and 2 of these cases appear to be deviant cases. The question is whether these are ‘true’ deviant cases or whether these are only ‘apparent’ deviant cases. Braumoeller and Goertz provide us a way to explore this possibility.

In this scenario, Braumoeller and Goertz argue we can test this in a very simple way.

First, we calculate the number of deviant cases as a proportion of all cases. In this scenario that would be 6.06%. We can call this $\hat{p}$ because it is an estimate of the proportion of deviant cases.

Second, we calculate a one-sided 95 confidence interval for $\hat{p}$ using a standard binomial distribution. This one-sided confidence interval (or lower bound) we can call 𝛼$.$In this instance 𝛼 = 0.0109.

Third, we calculate (or estimate) the error rate in the data (call this ε). This is often unknown – as is the case in our study.

Fourth, we compare the two statistics. The decision rule is:

1. We reject the hypothesis of necessity if 𝛼 > ε.
2. We fail to reject the hypothesis if 𝛼 < ε.

In other words, if error rate of the data (ε) is lower than the lower bound of the confidence interval around the number of deviant cases (𝛼), then we can be confident that our treatment variable is not a necessary condition. The value of this test is that it guards against situations in which the number of counterexamples is high and the estimated error is low.

In our case, this lower bound is 0.0109 (𝛼), which means we know with 95% certainty that the population proportion of cases in this cell is greater than 1.09%. This is a small number of counterexamples. According to the decision rule above, we can reject Braumoeller and Goertz’s p_I_-test of necessity if there are potentially more counterexamples in our data than could be accounted for by measurement error. For example, if the error rate was 3% then we could *not* reject the hypothesis of necessity because the error rate is higher than the number of counterexamples and these counterexamples could therefore be measurement error. In contrast, if the error rate was less than 1% then we could reject the hypothesis of necessity because the number of counterexamples is higher than the error rate.

In our data, we would need to a very high degree of confidence that there is no measurement error in our data (less than 1% of cases would have to be misclassified) for us to conclude that our deviant cases are “true” counter examples and that protest-led transitions are not a necessary condition for healthy democratic transitions.

The crucial implication here is that if we need to assume a very high level of measurement accuracy to be confident that the condition of necessity is *not* met. This leads us to fail to reject the hypothesis if necessity.
